# COVID surveillance robot: Monitoring social distancing constraints in indoor scenarios

**DOI:** 10.1371/journal.pone.0259713

**Published:** 2021-12-01

**Authors:** Adarsh Jagan Sathyamoorthy, Utsav Patel, Moumita Paul, Yash Savle, Dinesh Manocha

**Affiliations:** 1 Department of Electrical and Computer Engineering, University of Maryland, College Park, Maryland, United States of America; 2 Department of Computer Science, University of Maryland, College Park, Maryland, United States of America; 3 Institute of Systems Research, University of Maryland, College Park, Maryland, United States of America; Universiti Sains Malaysia, MALAYSIA

## Abstract

Observing social/physical distancing norms between humans has become an indispensable precaution to slow down the transmission of COVID-19. We present a novel method to automatically detect pairs of humans in a crowded scenario who are not maintaining social distancing, i.e. about 2 meters of space between them using an autonomous mobile robot and existing CCTV (Closed-Circuit TeleVision) cameras. The robot is equipped with commodity sensors, namely an RGB-D (Red Green Blue—Depth) camera and a 2-D lidar to detect social distancing breaches within their sensing range and navigate towards the location of the breach. Moreover, it discreetly alerts the relevant people to move apart by using a mounted display. In addition, we also equip the robot with a thermal camera that transmits thermal images to security/healthcare personnel who monitors COVID symptoms such as a fever. In indoor scenarios, we integrate the mobile robot setup with a static wall-mounted CCTV camera to further improve the number of social distancing breaches detected, accurately pursuing walking groups of people etc. We highlight the performance benefits of our robot + CCTV approach in different static and dynamic indoor scenarios.

## Introduction

The COVID-19 (COrona VIrus Disease-19) pandemic has caused significant loss of life around the world due to its high infection rate. One of the best ways to prevent contracting COVID-19 is to avoid being exposed to the SARS-CoV-2 virus. Organizations such as the Centers for Disease Control and Prevention (CDC) have recommended many guidelines including maintaining social or physical distancing, wearing masks or other facial coverings, and frequent hand washing to reduce the chances of contracting or spreading the virus. Broadly, social distancing refers to the measures taken to reduce the frequency of people coming into contact with others, particularly by maintaining at least 2 meters (6 feet) of physical distance between individuals. Several groups have simulated the spread of the virus and shown that social distancing can significantly reduce the total number of infection cases [1–6].

Since social distancing is a fundamental method to tackle any pandemic, it is crucial to develop technologies to help detect scenarios where such rules are not being followed, so that appropriate counter-measures can be employed. These actions could also help with collecting data about people who have come in contact with an infected person (contact tracing) [6]. Such technologies would also be general enough to help tackle the next pandemic with relative ease.

A comprehensive survey of all the technologies that can be used to detect if social distancing norms are followed properly is given in [6]. This includes a discussion of the pros and cons of technologies such as WiFi (WIreless FIdelity), Zigbee, RFID (Radio Frequency Identification), Cellular, Bluetooth, Computer Vision, AI (Artificial Intelligence), etc. However, many of these technologies require new static, indoor infrastructure such as WiFi routers, Bluetooth modules, central RFID hubs, etc or people to wear detectable tags. Technologies such as WiFi and Bluetooth require people to connect to them using wearable devices or smartphones for tracking. This limits their usage for tracking crowds and social distancing norms in general environments or public places, and may hinder the use of any kind of counter-measures.

Recently, there have also been several vision-based methods that use deep learning [7, 8] to detect social distancing breaches. A large-scale social distancing monitoring system using networked cameras has also been proposed [9]. Solutions based on semi-autonomous and autonomous robots [10] are also becoming popular. For instance, in [11], a quadruped robot with multiple on-board cameras and a 3-D lidar is used to enforce social distancing in outdoor crowds using sound-based alerts. Our work is complementary to these methods and also helps react to social distancing violations.

Another important component of our work is robot navigation amongst humans and other obstacles, which requires excellent collision avoidance capabilities. The problem of collision-free navigation has been extensively studied in robotics and related areas. Recently, some promising methods for navigation using noisy sensor data based on Deep Reinforcement Learning (DRL) methods [12, 13] have emerged. These methods produce better empirical results when compared to traditional methods [14–16].

Methods such as [17] train a decentralized collision avoidance policy by using raw data from a 2-D lidar, the robot’s odometry, and the relative goal location. Other works have developed learning-based policies that implicitly fuse data from multiple perception sensors to handle occluded spaces [18] and to better handle the Freezing Robot Problem (FRP) [19]. Other hybrid learning and model-based methods include [20], which predicts the pedestrian movement through optical flow estimation. In this work, we use Frozone [21], a state-of-the-art hybrid collision avoidance method, which is a combination of a DRL-based method and a model-based method (refer Frozone subsection).

### Main results

**Fig 1:**
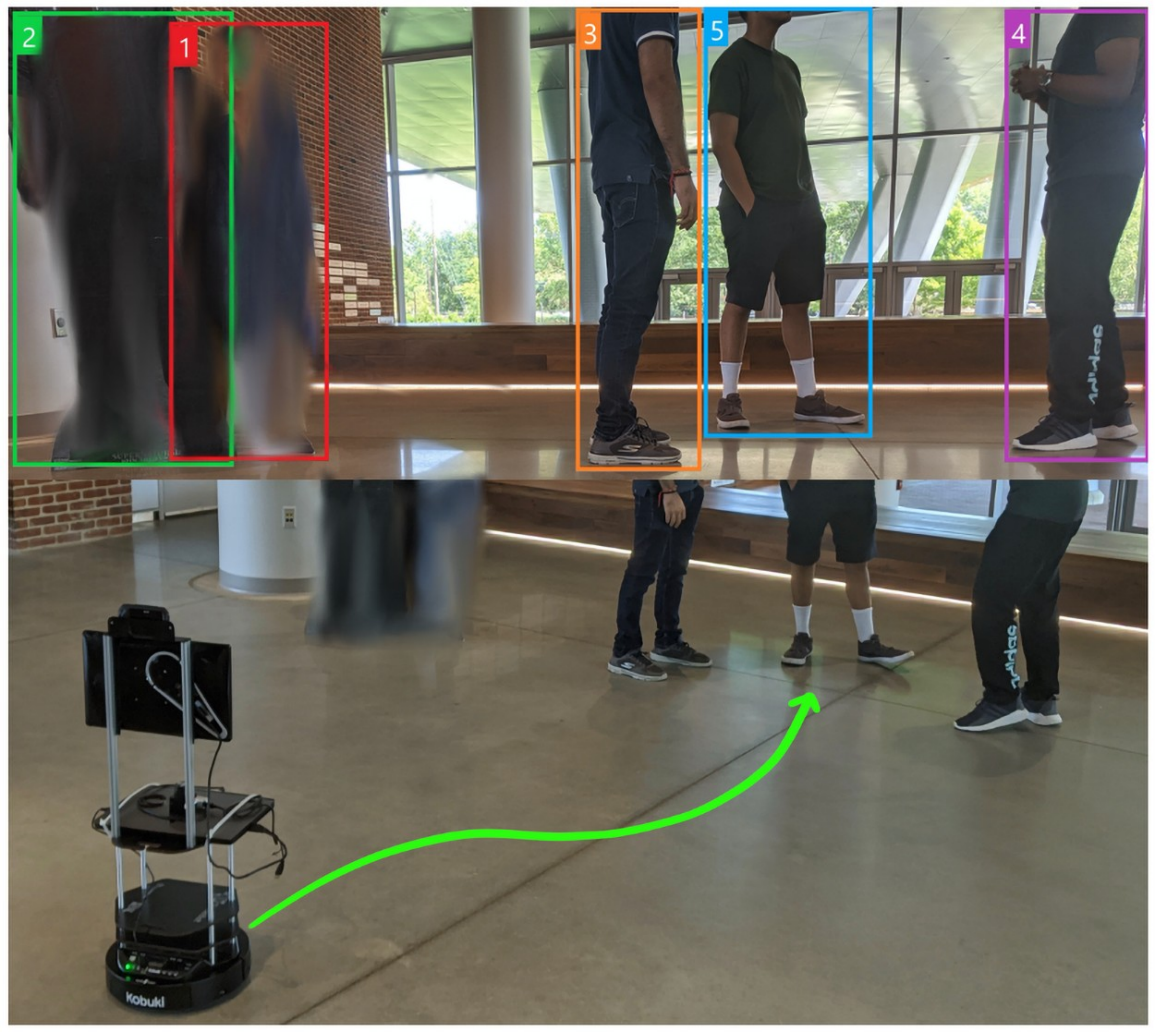
CS-robot detecting social distancing breaches. Our robot detecting non-compliance to social distancing norms, classifying non-compliant pedestrians into groups and autonomously navigating to the static group with the most people in it (a group with 3 people in this scenario). The robot encourages the non-compliant pedestrians to move apart and maintain at least 2 meters of social distance by displaying a message on the mounted screen. Our CS-robot also captures thermal images of the scene and transmits them to appropriate security/healthcare personnel.

We present a method for employing a vision-guided COVID Surveillance robot (CS-robot) and wall-mounted CCTV cameras (if available) to monitor scenarios with low to medium-density crowds to detect prolonged contact between individuals (Fig 1). We refer to such scenarios as social distancing breaches or just breaches in this work. Once a breach is detected, in static scenarios, the robot prioritizes *non-compliant* groups of people based on their size, autonomously navigates to the largest group and encourages them to follow the social distancing norms by displaying an alert message on a mounted screen. In dynamic scenarios, the robot prioritizes attending to groups depending on their motions relative to the robot.

We use Frozone [21] for the robot to avoid collisions and autonomously navigate towards non-compliant groups of people. CS-robot uses relatively inexpensive visual sensors such as an RGB-D camera and a 2-D lidar to provide inputs to Frozone for navigation, as well as to pedestrian detection and tracking algorithms which detect and classify pedestrians that violate social distance constraints as *non-compliant* pedestrians.

In indoor scenarios, our method integrates CS-robot with a CCTV camera setup (if available) to further improve its breach detection accuracy and monitor a larger area for social distancing breaches. A thermal camera, mounted on the robot is used to wirelessly transmit thermal images to healthcare personnel, which helps with symptom monitoring.

Our main contributions in this work are:

1. An automatic approach that can use a mobile wheeled robot system (CS-robot) and wall-mounted CCTV cameras (if available) to detect breaches in social distancing norms. We use a real-time method to estimate distances between people in images captured using an RGB-D camera on the robot and RGB images captured by the CCTV camera. The robot then enforces social distancing by navigating towards the groups of *non-compliant* people, and encouraging them to maintain at least 2 meters of distance by displaying an alert message discreetly on a mounted screen. Our overall system’s architecture allows the robot to detect and enforce breaches regardless of the availability of CCTV cameras in indoor settings. In the absence of CCTV cameras, we demonstrate that our robot monitoring system is effective in detecting breaches and can enforce social distancing in all the cases where breaches are detected. When a CCTV camera is available, the robot-CCTV hybrid setup significantly increases the area being monitored and improves the accuracy of tracking and navigating to dynamic non-compliant pedestrians. We demonstrate how simple techniques such as homography transformations could be used in the images from the CCTV camera for distance estimation between people. This hybrid combination of static mounted cameras and a mobile robot leads to the best results, and improves the number of breaches detected and enforcements by up to 100%. Furthermore, our approach does not require the humans to wear any tracking or wearable devices.
2. A novel method to classify non-compliant people into different groups, prioritize attending to them based on whether they are static or dynamic, and compute a goal for the robot to navigate towards the group. In static scenes, the goal is computed such that the robot attends the largest group first. In dynamic scenes, the goal computation depends on the motion of the groups relative to the robot. Our method intelligently locks on to a person in a dynamic non-compliant group to more accurately pursue it. We also deploy appropriate measures for privacy protection and de-identification.

We evaluate our method quantitatively in terms of accuracy of localizing a pedestrian in both static and dynamic environments. We highlight the number of social distancing breaches detected and attended, while using the different isolated components of our CCTV-robot hybrid system. We also measure the time duration for which the robot can track a dynamic pedestrian. Qualitatively, we highlight the trajectories of the robot pursuing dynamic pedestrians when using only its RGB-D sensor as compared to when both the CCTV and RGB-D cameras are used.

## Methods

### Background

In this section, we provide a brief overview of the pedestrian detection and tracking method and the collision avoidance scheme that we use. We also describe our criteria for a social distancing breach.

#### Pedestrian detection and tracking

For detecting and tracking pedestrians, we use the work done in [22] based on Yolov3 (You Only Look Once version 3) [23], which achieves a good balance between speed and tracking accuracy. The input to the tracking scheme is an RGB image (from an RGB-D or an available CCTV camera) and the output is a set of corner coordinates of the bounding boxes (denoted as B) for all the pedestrians detected in the image.

Yolov3 also outputs a unique ID for every person in the RGB image, which remains constant as long as the person remains in the camera’s FOV (Field Of View). Based on a person’s bounding box locations in two images, and the time interval between the two images, a person’s walking speed and direction (and therefore the walking vector) can be trivially estimated.

#### Frozone

To navigate a robot towards a goal in the presence of humans, we use Frozone [21], a state-of-the-art collision avoidance method that uses an RGB-D camera to track and predict the future positions and orientations of pedestrians relative to the robot. For these computations, Frozone uses the outputs from the pedestrian detection and tracking module. When navigating among humans, Frozone minimizes the occurrence of the robot halting/freezing, as it severely affects its navigation and causes obtrusion to the humans around it.

The main goal of Frozone is to avoid a space called the *Potential Freezing Zone* (PFZ), where the robot has a high probability of freezing and being obtrusive to pedestrians after a time interval Δ*t*. It is constructed as,

$$\begin{array}{c} PFZ=ConvexHull({\hat{p}}_{i}^{ped}),\;i\in 1,2,\ldots ,K. \end{array}$$ (1)

Where ${\hat{p}}_{i}^{ped}$ is the predicted future position of the *i*^*th*^ pedestrian, and K is the total number of pedestrians that might cause the robot to halt/freeze. *ConvexHull()* denotes the convex hull function. If the robot is heading towards a PFZ while proceeding towards its goal, Frozone computes a deviation angle to avoid it.

#### Criteria for social distancing breach

**Fig 2:**
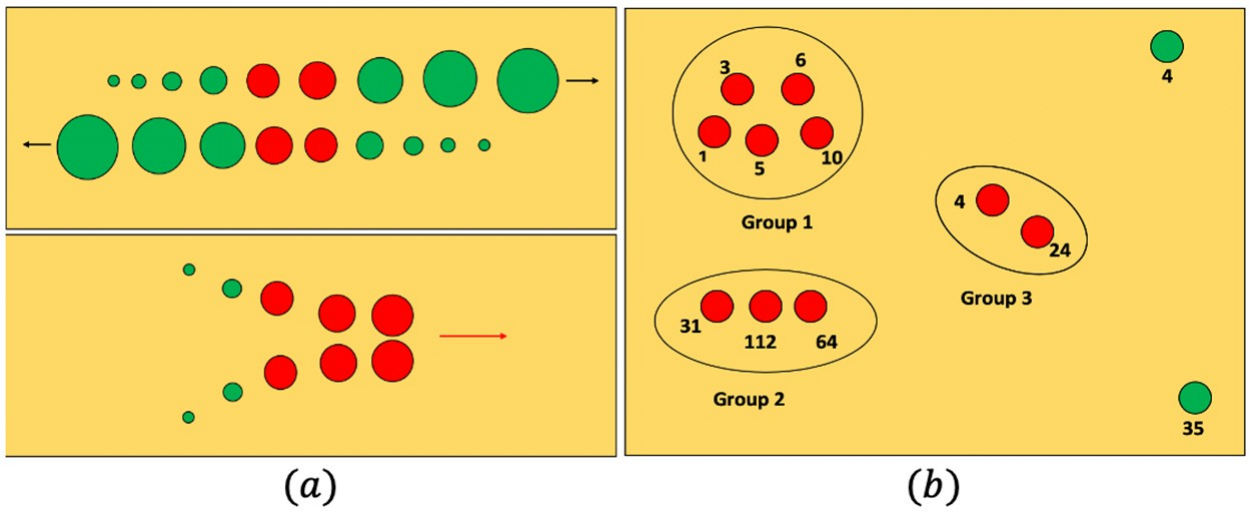
Criteria for a social distancing breach. **(a)**: Our criteria used to detect whether two pedestrians violate the social distance constraint. The pedestrians are represented as circles in two different scenarios. The increasing size of the circles denotes the passage of time. The green circles represent time instants when the pedestrians maintained > 2 meters distance, and the red circles represent instants when they were closer than 2 meters. **Top**: Two pedestrians passing each other. This scenario is not reported as a breach since the duration of the breach is short. **Bottom**: Two pedestrians meeting and walking together. This scenario is reported as a breach of social distancing norms. **(b)**: A top-down view of how non-compliant pedestrians (denoted as red circles) are classified into groups. The numbers beside the circles represent the IDs of the pedestrians outputted by Yolov3. The compliant pedestrians (green circles) are not classified into groups as the robot does not have to encourage them to maintain the appropriate social distance.

We mainly focus on detecting scenarios where individuals do not maintain a distance of at least 2 meters from others *for a given period of time*. We choose to detect this scenario because it is a fundamental social distancing norm during all stages of a pandemic, even as people begin to use public spaces and restrictions are lifted. An important challenge is to avoid detecting two or more people passing each other as a breach, even if the distance between them was less than 2 meters for a few moments (see Fig 2a).

### Breach detection

**Fig 3:**
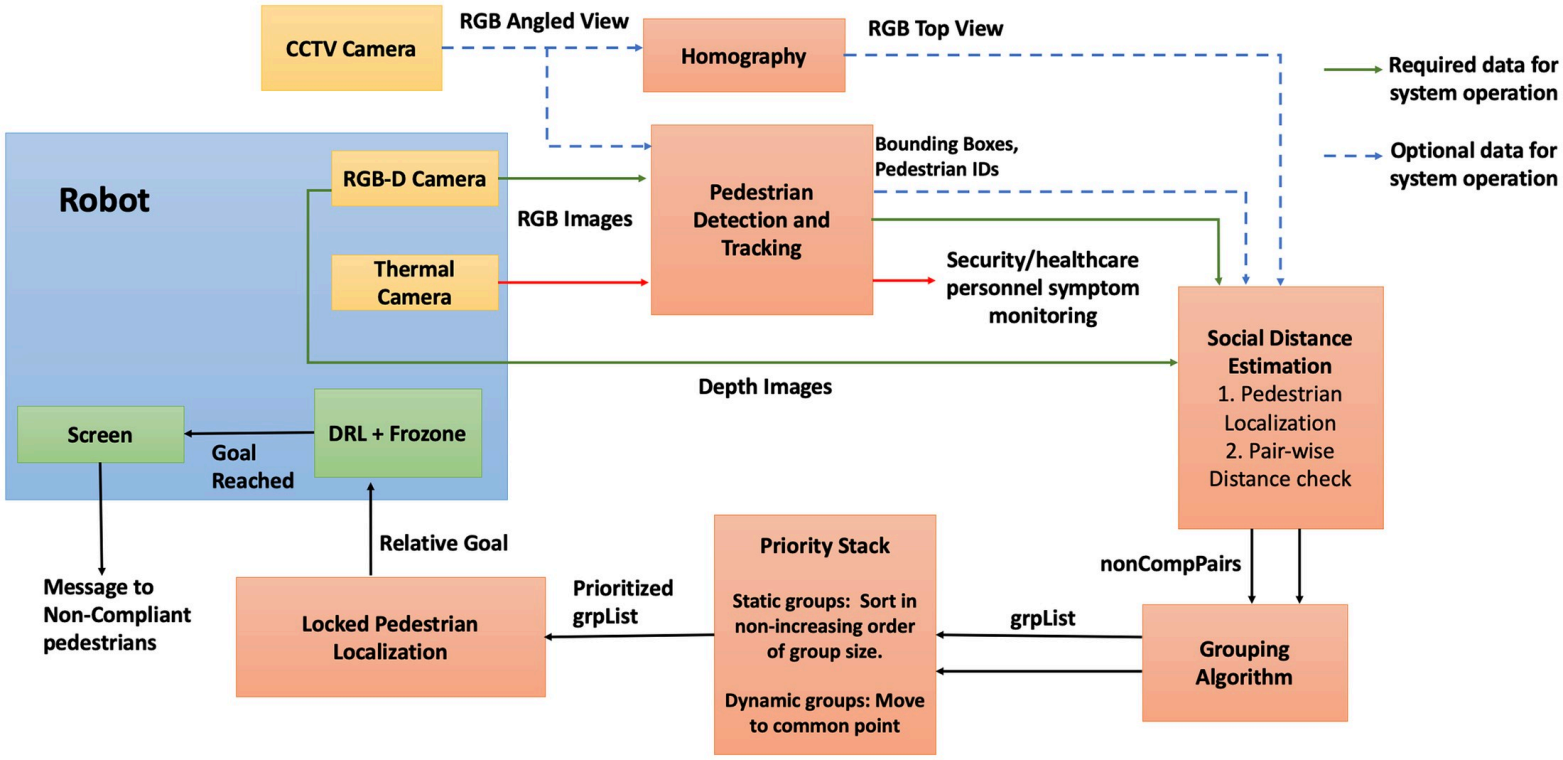
Overall system architecture. Overall architecture of social distance monitoring using CS-robot: The main components include: (i) Pedestrian tracking and localization; (ii) Pairwise distance estimation between pedestrians; (iii) Classifying pedestrians into groups; (iv) Computing a goal for the robot based on whether the group is static or dynamic; (v) Using a hybrid collision avoidance method to navigate towards the goal; (vi) Displaying an alert message to the non-compliant pedestrians to encourage them to move apart; (vii) Thermal image and bounding boxes of detected people are transmitted to security/healthcare personnel.

In this section, we first explain the architecture of our robot and CCTV setup and the relationship between all the sensors used in the system. We then describe how our method effectively detects a social distancing breach. We refer to people who violate social distancing norms as *non-compliant* pedestrians. We then describe how we classify non-compliant pedestrians into groups and compute the goal for the robot’s navigation for static and dynamic scenarios. Our overall system architecture is shown in Fig 3.

#### System architecture

Our overall system architecture is shown in Fig 3. Our architecture contains three cameras: 1. RGB-D camera on-board the robot, 2. CCTV camera mounted on the wall (if available), and 3. Thermal camera on-board the robot. The images from the RGB-D (green arrows) and CCTV cameras (blue dashed arrows in Fig 3) are used as inputs to various modules such as pedestrian tracking, homography, and eventually social distance estimation. The data represented by the green arrows is required, whereas the data represented by the blue arrows is optional for the overall operation of the system. The thermal image (red line in Fig 3) is analyzed by the pedestrian detection and tracking module, and the outputs are sent only to security or healthcare personnel to monitor symptoms of the tracked people (see Thermal Camera section). It is not used for social distance estimation or aiding the robot in navigation.

The architecture is designed in a way that the robot can detect social distancing breaches and navigate to non-compliant groups independent of the availability of the CCTV camera using only its on-board RGB-D camera. In the presence of a CCTV camera, the architecture uses its images to more effectively detect non-compliant groups in a larger sensing region with a global perspective and guide the robot.

We detect breach scenarios based on the criteria previously mentioned. The robot’s on-board RGB-D camera and the CCTV camera setup (whenever available) continuously monitor the states of individuals within their sensing range. At any instant, breaches could be detected by the robot’s RGB-D camera and/or the CCTV camera.

#### Social distance estimation using RGB-D camera

At any time instant *t*, we first localize a person detected in the RGB image relative to the robot by using the depth image *I*^*t*^. The depth image is calibrated to be the same shape as the RGB image. Every pixel in *I*^*t*^ contains the proximity of an object at that location of the image.

**Fig 4:**
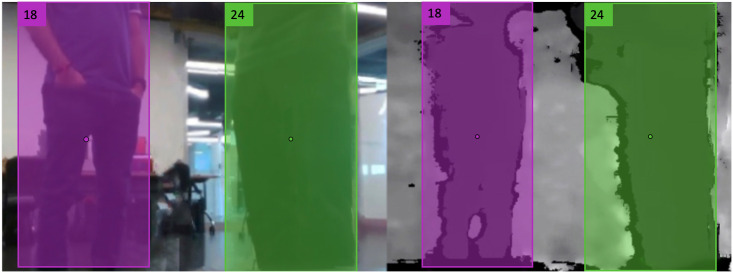
Pedestrian localization using RGB-D camera. **Left**: Two pedestrians detected in the RGB image of the robot’s RGB-D camera with the bounding box centroids marked in pink and green. **Right**: The same bounding boxes superimposed over the depth image from the RGB-D camera. The pedestrians are localized and the distance between them is estimated by the method detailed above.

First, the detection bounding boxes from the RGB image passed through the pedestrian tracking method are superimposed over the depth image (Fig 4). Next, the minimum 10% of the pixel values inside the bounding box $B_{P}$ are averaged to obtain the mean distance ($d_{rob}^{P}$) of a pedestrian *P* relative to the robot. Denoting the centroid of the bounding box $B_{P}$ as $\left[x_{cen}^{B_{P}},y_{cen}^{B_{P}}\right]$, the angular displacement $\psi_{rob}^{P}$ of the pedestrian relative to a coordinate frame attached to the robot (with X-axis and Y-axis pointing in the forward and left directions of the robot, respectively) can be computed as:

$$\begin{array}{c} \psi_{rob}^{P}=(\frac{\frac{w}{2}-x_{cen}^{B_{P}}}{w})*FOV_{RGBD}. \end{array}$$ (2)

Here *FOV*_*RGBD*_ is the field of view angle of the RGB-D camera and *w* is the width of the depth image. We note that $\psi_{rob}^{P}\in \left[-FOV_{RGBD}/2,\;\;FOV_{RGBD}/2\right]$. The pedestrian’s position with respect to the robot is then calculated as $\left[x_{rob}^{P}\;\;y_{rob}^{P}\right]=d_{rob}^{P}*[\text{cos}\;\psi_{rob}^{P}\;\;\text{sin}\;\psi_{rob}^{P}$]. To estimate the distances between a pair of pedestrians, say *P*_*a*_ and *P*_*b*_, we use the Euclidean distance function given by,

$$\begin{array}{c} dist\left(P_{a},P_{b}\right)=\sqrt{{\left(x_{rob}^{P_{a}}-x_{rob}^{P_{b}}\right)}^{2}+{\left(y_{rob}^{P_{a}}-y_{rob}^{P_{b}}\right)}^{2}.} \end{array}$$ (3)

#### Social distance estimation using a CCTV camera

Although the RGB-D camera is mobile, it is limited by a low FOV and sensing range. A breach occurring outside its sensing range will not be reported. Therefore, we utilize an existing CCTV camera setup in indoor settings to widen the scope for detecting breaches. Pedestrian detection and tracking are done as described in the Background section. We estimate distances between individuals as follows.

**Fig 5:**
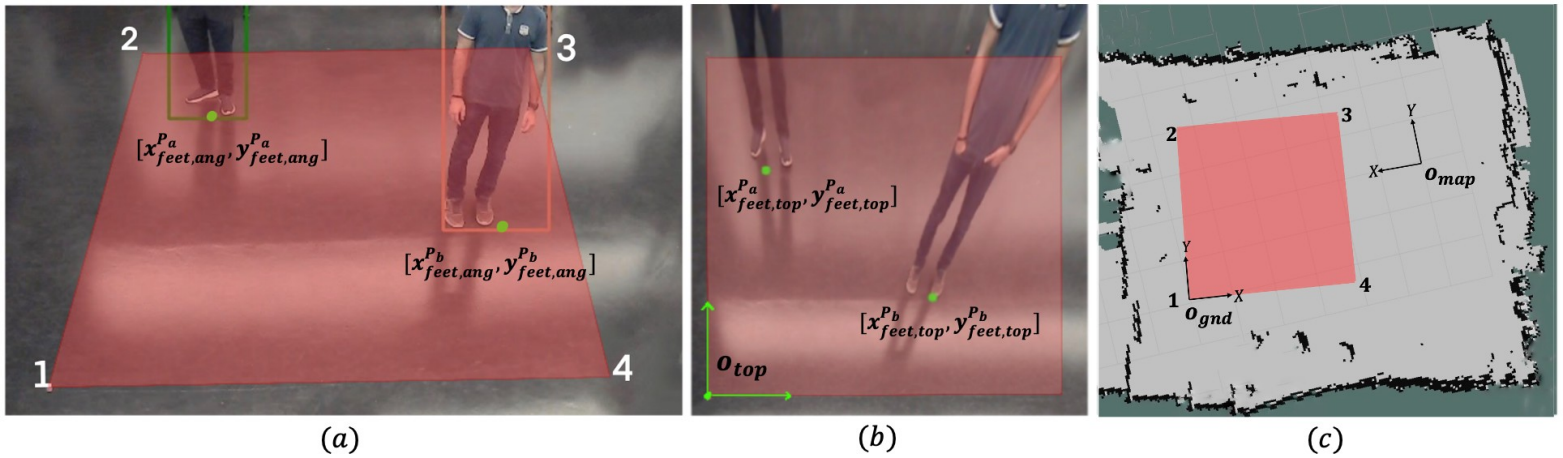
Homography and coordinate frames. **a**. The angled view of the homography rectangle marked in red and corners numbered from the CCTV camera. The green dots mark the points corresponding to a person’s feet in this view. **b**. The top view of the homography rectangle after transformation and the origin of the top view coordinate system is marked as *o*_*top*_. The coordinates of the feet points are also transformed using the homography matrix. **c**. A map of the robot’s environment with free space denoted in gray and obstacles denoted in black with a coordinate frame at origin *o*_*map*_. The homography rectangle is marked in red and the ground plane coordinate system is shown with the origin *o*_*gnd*_.

*Homography*. All CCTV cameras are mounted such that they provide an *angled* view of the ground plane. However, to accurately calculate the distance between any two people on the ground, a top view of the ground plane is preferable. To obtain the top view, we apply a homography transformation to four points on the ground plane in the angled view that form the corners of the maximum-area rectangle that can fit within the FOV of the CCTV camera (see Fig 5a and 5b) as,

$$\begin{array}{c} {\left[x_{corn,top}\;\;y_{corn,top}\;\;1\right]}^{T}=M*{\left[x_{corn,ang}\;\;y_{corn,ang}\;\;1\right]}^{T}. \end{array}$$ (4)

Here, *x*_*corn*,*ang*_ and *y*_*corn*,*ang*_ denote the pixel coordinates of one of the four points in the angled CCTV view image. We call the rectangle in the top view, the homography rectangle. *x*_*corn*,*top*_ and *y*_*corn*,*top*_ denote the same point in the transformed top view, and M is the scaled homography matrix which is computed using standard OpenCV (Open Source Computer Vision Library) functions.

*Distance estimation between pedestrians*. We localize each detected pedestrian within the homography rectangle as follows. We first obtain a point corresponding to the feet of a pedestrian P (denoted as [$x_{feet,ang}^{P},y_{feet,ang}^{P}$]) by averaging the coordinates of the bottom left and the bottom right corners of the bounding box of the pedestrian (see Fig 5a) in the angled CCTV view. This point is then transformed to the top view using Eq 4 as ${\left[x_{feet,top}^{P},y_{feet,top}^{P}\right]}^{T}=M*{\left[x_{feet,ang}^{P},y_{feet,ang}^{P}\right]}^{T}$ (see Fig 5b).

The distance between any two pedestrians *P*_*a*_ and *P*_*b*_ is first calculated by using Eq 3 with the coordinates [$x_{feet,top}^{P_{a}},y_{feet,top}^{P_{a}}$] and [$x_{feet,top}^{P_{b}},y_{feet,top}^{P_{b}}$] which is then scaled by an appropriate factor S to obtain the real-world distance between the pedestrians. The scaling factor is found by measuring the number of pixels in the image that constitute 1 meter in the real-world.

If the real-world distance estimated from the RGB-D or the CCTV camera images between a pair of pedestrians is less than 2m for a period of time T, a breach is reported for that pair. This process is continued in a pairwise manner and a list of all the pairs of non-compliant pedestrian IDs is then obtained.

### Enforcing social distancing

Once breaches are detected, either by the RGB-D camera or the CCTV camera or both, the robot must first prioritize them, and compute a goal location in the vicinity of the top-priority breach relative to itself. It must then navigate towards this goal and encourage the non-compliant pedestrians to move apart by displaying an alert message discreetly. We detail the steps involved in the following sections.

#### Classifying people into groups

In social scenarios, people naturally tend to walk or stand in groups. We define a group as a set of non-compliant people who are closer than 2 meters from each other (see Fig 2b). We assume that if the robot reaches the vicinity of a group, it can alert everyone in that group to observe social distancing. In addition, when there are multiple groups of non-compliant people, the robot can prioritize approaching different groups based on whether they are static or dynamic. We classify non-compliant people into groups based on Algorithm 1.

**Algorithm 1**: Non-Compliant Group Classification.

**Input**: A list nonCompPairs of length *S*_*input*_

**Output**: A list grpList

1: nonCompPairs ← List of pedestrian ID pairs breaching social distancing

2: grpList ← nonCompPairs [0]

3: **for** i from 1 to *S*_*input*_ **do**

4:  counter ←0

5:  **for** j from 0 to len(grpList) **do**

6:   intersection ← grpList[j] ∩ nonCompPairs[i]

7:   **if** intersection ≠ ∅ **then**

8:    grpList[j] ← grpList[j] ∪ grpList[i]

9:   **else**

10:    counter ← counter + 1

11:   **end if**

12:  **end for**

13:  **if** counter == *len*(grpList) **then**

14:   grpList.append(nonCompPairs[i])

15:  **end if**

16: **end for**

In Algorithm 1, nonCompPairs is a list that contains the IDs of all the pairs of non-compliant pedestrians obtained in the Breach Detection section. grpList is a list of groups where each group contains the IDs of people who have been assigned to it.

#### Locked pedestrian

To navigate towards a non-compliant group of people, the RGB-D or the CCTV camera must be able to track at least one member of that group to compute a goal in its vicinity. Our method locks on to a person with the least probability of exiting the FOV of either the RGB-D or the CCTV camera (depending on which camera detected the group). This person is called the *locked pedestrian*, and their ID is updated as people’s positions change in the cameras’ FOVs. The person whose bounding box centroid has the least lateral distance from the center of the image is chosen as the locked pedestrian. The condition is,

$$\begin{array}{c} x_{cen}^{B_{lp,i}}-\frac{w}{2}=\underset{P\in I_{G_{i}}}{\text{min}}x_{cen}^{B_{P}}-\frac{w}{2}, \end{array}$$ (5)

where $I_{G_{i}}$ is the set of IDs for the detected pedestrians and $B_{lp_{i}}$ denotes the bounding box of the locked pedestrian in the *i*^*th*^ non-compliant group inside the FOV.

#### Prioritizing groups in different scenarios

When multiple static and dynamic non-compliant groups are present, our method prioritizes attending to them based on whether the groups are (i) entirely or partially static, or (ii) entirely dynamic.

*Entirely or partially static groups*. When all or some of the groups within the FOV are static, our method prioritizes attending to each static group by sorting them in the non-increasing order of the number of people in group. Our method then computes a goal to which the robot will navigate based on the locked pedestrian’s location in each group.

If only the RGB-D is in use, the robot localizes the locked pedestrian in the highest priority group relative to itself using Eq 2). That is, $\left[x_{goal,rob}\;\;y_{goal,rob}\right]=d_{rob}^{lp}*{\left[\text{cos}\;\psi_{rob}^{lp}\;\;\text{sin}\;\psi_{rob}^{lp}\right]}^{T}$.

Where, [*x*_*goal*,*rob*_ *y*_*goal*,*rob*_] is the goal location relative to the robot, $d_{avg}^{lp}$ and $\psi_{rob}^{lp}$ are the average distance and angular displacement of a locked pedestrian from the robot respectively.

If a CCTV camera is available, the goal computation for the robot requires homogeneous transformations between three coordinate frames: 1. the top-view image obtained after homography, 2. the ground plane, and 3. a map of the environment in which the robot is localized. These three coordinates with origins *o*_*top*_, *o*_*gnd*_ and *o*_*map*_ respectively are shown in Fig 5b and 5c. A detailed explanation of our goal calculation can be found in [24]. We provide the final result here:

$$\begin{array}{c} \left[x_{goal,rob}\;\;y_{goal,rob}\right]={\left[x_{feet,map}^{lp}\;\;y_{feet,map}^{lp}\right]}^{T}-{\left[x_{rob,map}\;\;y_{rob,map}\right]}^{T}. \end{array}$$ (6)

Where *x*_*rob*,*map*_, *y*_*rob*,*map*_ and $x_{feet,map}^{lp}$, $y_{feet,map}^{lp}$ are the X and Y coordinates of the robot and the locked pedestrian’s feet in the map coordinate frame, respectively.

*Dynamic groups*. When all the groups in the FOV are dynamic, we use an optimization formulation to compute a goal to attend to the *greatest number of people* before they move away. We first differentiate the groups that are moving closer to and away from the robot by checking if,

$$\begin{array}{c} \begin{array}{c} \sqrt{{\left({\hat{x}}_{rob}^{lp}\right)}^{2}+{\left({\hat{y}}_{rob}^{lp}\right)}^{2}}\leq \sqrt{{\left(x_{rob}^{lp}\right)}^{2}+{\left(y_{rob}^{lp}\right)}^{2}},\\ {}[{\hat{x}}_{rob}^{lp}\;\;{\hat{y}}_{rob}^{lp}{]}^{T}={\left[x_{rob}^{lp}\;\;y_{rob}^{lp}\right]}^{T}+v_{rob}^{lp}\Delta t \end{array} \end{array}$$ (7)

where $\left[x_{rob}^{lp}\;\;y_{rob}^{lp}\right]$ and $\left[{\hat{x}}_{rob}^{lp}\;\;{\hat{y}}_{rob}^{lp}\right]$ denote the current position and position after a small time interval Δ*t* of a locked pedestrian of a group relative to the robot. $v_{rob}^{lp}$ is the estimated walking vector of the locked pedestrian relative to the robot (as explained in the pedestrian detection section). For all the groups that satisfy this condition, we compute the robot’s goal as,

$$\begin{array}{c} \left[x_{goal,rob}\;\;y_{goal,rob}\right]=\underset{x,y}{\text{argmin}}\sum_{i=1}^{N}dist\left(\left[x,y\right],\left[{\tilde{x}}_{rob}^{lp},{\tilde{y}}_{rob}^{lp}\right]\right) \end{array}$$ (8)

where N is the number of groups that satisfy the condition in Eq 7. This goal is a common point from which all the groups moving towards the robot can be alerted. We note that this formulation is effective for a small number of groups within the FOV, which we assume is the case when pandemic restrictions are in place. When *N* = 1, the robot actively pursues the group to alert them to maintain social distancing.

#### Multiple groups, lawnmower inspection and alerting

If the same group is detected in both cameras, the goal computed using the CCTV camera is used to guide the robot. The robot uses a display mounted on it to convey a message encouraging social distancing discreetly, once it reaches a group. To improve the effectiveness of the integrated robot and CCTV system in detecting new non-compliant groups of pedestrians, the robot inspects the blind spots of the CCTV camera continuously by following the well-known lawnmower strategy. In addition, the lawnmower strategy guarantees that 100% of an environment can be covered by navigating to a few fixed waypoints, although it does not guarantee an increase in the number of breaches detected.

### Thermal camera and privacy

**Fig 6:**
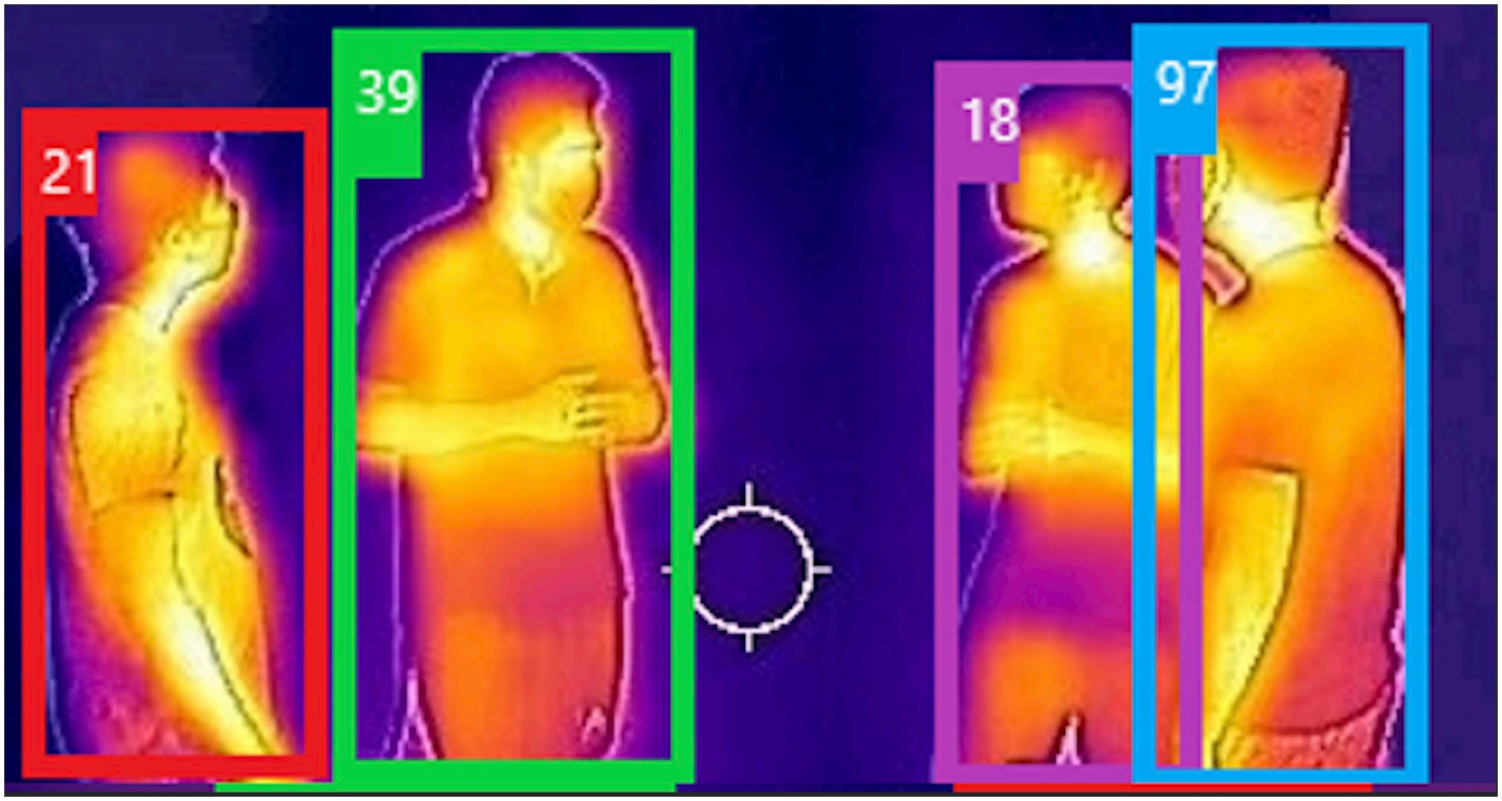
Thermal camera images. Thermal images generated by the thermal camera that is wirelessly transmitted to appropriate security/healthcare personnel. The temperature signatures of the people remain constant irrespective of their orientations. We intentionally have a human in the loop to monitor people’s temperature signatures, and we do not perform any form of facial recognition on people to protect their privacy. Pedestrians are detected on the thermal image to aid the personnel responsible for monitoring the area.

As mentioned before, we use the thermal camera exclusively for COVID-19 symptom monitoring. We use our pedestrian detection scheme to detect and track people in the thermal images (see Fig 6) and the results are then sent to appropriate security or healthcare personnel who check for COVID-19 symptoms such as a fever. Such a system would be useful in places where people’s temperatures are already measured by security/healthcare personnel such as airports, hospitals etc. Monitoring people’s temperatures remotely reduces the risk of the security/healthcare personnel contracting the coronavirus while also protecting people’s privacy. In addition, we ensure that our algorithms do not use any identifiable information such as facial features.

### Ethics and privacy

All the evaluations in the following section were conducted based on an IRB (Institutional Review Board) approval by the University of Maryland Institutional Review Board Office (IRB #1608506–1). We followed acceptable privacy preserving guidelines in protecting people’s private health information. In particular, we ensure the following: no identifiable information (such as face) of a person is detected, nor stored on any server. Thermal image data is collected only for knowing if a person with symptoms has been in a certain region so appropriate countermeasures can be employed. It is not linked to the person’s age, gender, name, race, ethnicity etc. We also use standard de-identification methods such as visual image redaction for faces, gesture and gait data. The University of Maryland Institutional Review Board Office waived the need for verbal or written consent for our study since we do not map people’s trajectories to any of their personal information. The individuals pictured in Figs 1 and 6 and Supporting information files (S1 Video) have provided written informed consent (as outlined in PLOS consent form) to publish their image alongside the manuscript.

## Results and discussion

In this section, we elaborate on our method’s implementation on a robot, explain the evaluation scenarios and the metrics used to evaluate it and analyze its effectiveness and limitations.

### Implementation

We implement our method on a Turtlebot 2 robot customized with additional aluminium rods to attach a 15-inch screen to display messages to the non-compliant pedestrians. We limit the robot’s maximum linear and angular velocities to 0.65 m/s and 1 rad/s respectively. The pedestrian detection and tracking algorithm is executed on a laptop with an Intel i9 8th generation CPU and an Nvidia RTX2080 GPU mounted on the robot. We use an Intel RealSense (with 70° FOV) RGB-D camera to sense pedestrians and a Hokuyo 2-D lidar (240° FOV) to sense other environmental obstacles. To emulate a CCTV camera setup, we used an RGB camera with a 1080p resolution mounted at an elevation of 4 meters, and a sensing region of 4m × 4m. We use a desktop machine with an Intel Xeon 3.6GHz × 8 processor, Nvidia Titan GPU and 32GB RAM to track pedestrians in the images from the CCTV camera. We use a FLIR C3 thermal camera to generate the temperature signatures of the robot’s surroundings.

### Evaluation scenarios

For our evaluations, we consider an indoor environment with a wall-mounted CCTV camera with a rectangular sensing region of 4 meters by 4 meters (see Fig 5c). The robot is made to operate (either be static, execute lawnmower exploration or autonomously navigate to a non-compliant group) outside the CCTV’s sensing region. The environment can have static or dynamic obstacles other than the humans. We sample the environment uniformly and obtain 40 locations where people can stand while creating scenario 1 and 2. Twenty of these locations are inside the CCTV’s sensing region and when the robot is static, approximately 10 sampled locations are within the RGB-D camera’s sensing region.

- **Scenario 1**: One person stands in different locations in the sensing range of the RGB-D camera and CCTV camera. The person is allowed to orient themselves in any random direction at the location they stand.
- **Scenario 2**: Two people stand together (< 2 meters distance between them) in one of the 40 uniformly sampled locations in the environment. The people can individually be facing in any arbitrary orientation. They could be standing side by side without occluding each other or one person could occlude the other in various degrees (see Table 1). The robot could either be static or executing the lawnmower exploration routine.
- **Scenario 3**: A person walks 5 meters in a direction that is perpendicular to the orientation of the robot. The person walks with different speeds for different trials. The robot is static and is only allowed to rotate in place. The CCTV camera is not used in this scenario.
- **Scenario 4**: A certain number of people walk in random directions in the environment for a period of 60 seconds. The number of people could vary between two and six. They could walk alone or as a group with someone else for the entirety of the 60 seconds. The constraints in this scenario are that the number of non-compliant groups (people walking together) remains constant, and the members of one group do not interact or join another group. The robot executes the lawnmower exploration routine, and the CCTV camera is used to detect breaches.
- **Scenario 5**: Two people walk as a group in a random direction in the environment. Their trajectories could be smooth or have sudden sharp turns. The robot is allowed to be fully mobile and pursue the walking group. The CCTV is used to detect the group and guide the robot to pursue the non-compliant group.

**Table 1:** Comparison of breach detection and enforcement.

Case 1: Static robot no occlusions			
**Metric**	**CCTV-only**	**Robot-only**	**Robot-CCTV Hybrid**
Number of breaches detected	20	10	30
Number of enforcements	NA	10	30
Case 2: Static robot with 50% occlusion			
Number of breaches detected	20	7	27
Number of enforcements	NA	7	27
Case 3: Static robot with >50% occlusion			
Number of breaches detected	20	3	23
Number of enforcements	NA	3	23
Case 4: Lawnmower exploration with 50% occlusions			
Number of breaches detected	20	20	40
Number of enforcements	NA	20	40
Case 5: Lawnmower exploration with >50% occlusions			
Number of breaches detected	20	20	40
Number of enforcements	NA	20	40

Comparison of three configurations in terms of detecting breaches in social distancing norms when two pedestrians are static in any one of 40 points in a laboratory setting. We observe that CCTV+robot configuration has the most number of breaches detected even when the robot is static and outside the CCTV’s sensing range. When the robot is following lawnmower waypoints outside of the CCTV’s FOV, it can detect a breach in any of the 20 locations that could not be detected by the CCTV camera, even with high levels of occlusion.

### Quantitative metrics

We use the following metrics to evaluate our method.

1. **Accuracy of pedestrian localization**: It refers to the difference between the ground truth location of a pedestrian and the location estimated using our method when using an RGB-D or a CCTV camera.
2. **Number of breaches detected**: The number of locations in an environment (with or without occlusions) at which a social distancing breach can be detected, given a total number of locations uniformly sampled from the environment.
3. **Number of enforcements**: The number of times the robot attended to a breach once it was detected (with or without occlusions).
4. **Tracking duration for a mobile pedestrian**: The time for which the robot can track a walking pedestrian. Longer tracking duration translates to more accurate goal computation and pursuit when dealing with one or more dynamic groups.
5. **Breach detection accuracy**: This metric is defined by the following equation,
  $$\begin{array}{c} \text{Breach}\;\text{detection}\;\text{accuracy}=\frac{\sum_{i=1}^{T}\frac{\text{Number}\;\text{of}\;\text{breaches}\;{\text{detected}}_{i}}{\text{Actual}\;\text{number}\;\text{of}\;{\text{breaches}}_{i}}}{T}, \end{array}$$ (9)
  where T is the total number of seconds for which the experiment is conducted, and i is an index denoting every second until T seconds.

Metrics 1 is used for evaluation in scenario 1. Metrics 2, and 3 are used for evaluation in scenario 2. Metric 4 is used for evaluation in scenario 3, and metric 5 is used with scenario 4.

### Analysis

We elaborate our observations and the inferences drawn from our experiments conducted in the different scenarios previously described, using the aforementioned metrics.

#### Accuracy of pedestrian localization

**Fig 7:**
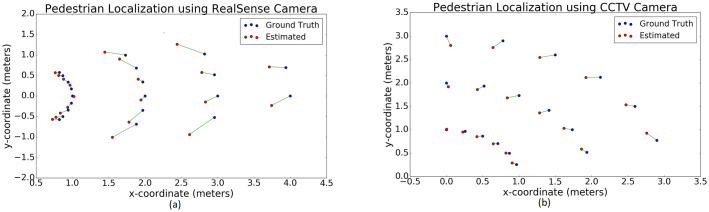
Localization accuracy results. Plots of ground truth (blue dots) versus estimated pedestrian localization (red dots) when using the robot’s RealSense camera and the static CCTV camera with more FOVs. **a**. The estimates from the RealSense camera tend to have slightly higher errors because we localize pedestrians using averaged proximity values within their detection bounding boxes, which is affected by the size of the bounding boxes. **b**. Localization using the data from the CCTV camera is more accurate as it tracks a person’s feet. This method is not affected by a person’s orientation. We observe that in both cases, the localization errors are within the acceptable range of 0.3 meters.

In scenario 1, we compare the ground truth locations with the estimated pedestrian location (metric 1) while using 1. the robot’s RGB-D camera, and 2. the CCTV setup. The plots are shown in Fig 7, with the ground truth locations plotted as blue circles and the estimated locations plotted as red circles. The plot in Fig 7a shows the pedestrian being localized relative to the robot coordinate frame (see the section on breach detection). Fig 7b shows a pedestrian being localized in the ground coordinate frame with origin **o**_*gnd*_, which corresponds to corner point 1 of the homography rectangle on the ground (see Fig 5c).

We observe in Fig 7a that when a pedestrian is located closer than 2.5 meters from the robot and along the robot’s X-axis, the localization estimates closely match the ground truth. The difference between ground truth and the estimate increases further away from the robot. This is primarily because the robot localizes a pedestrian using the minimum 10% of the pixels within his/her’s bounding box, whereas the ground truth is measured as a point on the ground.

Furthermore, the orientation of the pedestrian relative to the RGB-D camera also affects the their bounding box’s size and centroid, and therefore the localization estimate. However, since the maximum error between the ground truth and estimated values is within 0.3 meters, its effect on the social distance calculation and goal selection for the robot is within an acceptable limit. The accuracy can also be improved with higher FOV depth cameras in the future.

From Fig 7b we see a trend similar to the plot in Fig 7a. The farther away a person is from the origin (*o*_*gnd*_), the greater the error between the ground truth and the pedestrian’s estimated location. This is due to the approximations in the homography in obtaining the top view from the angled CCTV view, which carries forward to computing [$x_{feet,top}^{P_{a}},y_{feet,top}^{P_{a}}$] (see explanations in pedestrian localization using CCTV camera). However, the maximum error between the estimates and ground truths is again within 0.25 meters. Since a pedestrian’s location is estimated by the point corresponding to his/her feet, errors due to the pedestrian’s orientation are less frequent. The average error in the distance estimation between pedestrians is ∼ 0.1 meters.

#### Breach detection and enforcement

In scenario 2, we compare the performance differences in detecting a social distancing breach and enforcing for three configurations: 1. CCTV only, 2. Robot only, and 3. Robot-CCTV hybrid system. Scenario 2 emulates social settings where people could be sitting or standing and interacting with each other in public spaces.

The enforcement capabilities of these systems when people are walking vary extensively. They depend on the initial orientation of the robot (and its sensors) and the peoples’ walking directions and speeds. Therefore, for quantitative comparison, we first standardize our detection and enforcement tests in static settings. We quantify the breach detection capabilities in dynamic settings in the breach detection accuracy section. We qualitatively analyze the detection and pursuit of walking pedestrians in the CCTV-guided walking locked pedestrian pursuit section.

The results are shown in Table 1. The CCTV-only configuration is capable of detecting breaches which occur in the 20 sampled locations which are within its FOV. It can also handle occlusions (even > 50%) between pedestrians and detect breaches better due to its global view of the environment. We observed that even with the partial visibility of a person’s limbs, the breach detection was accurately performed. The random orientations of the people also do not affect the breach detection’s accuracy. We note that this system already improves over current CCTV systems where a human manually has to detect such breaches and initiates countermeasures. However, there is no scope for automatically enforcing social distancing at the location of the breaches.

The robot-only configuration detects fewer breaches than the CCTV setup when the robot is static (due to the RGB-D camera’s low FOV). Additionally, occlusions severely affect the robot’s number of detections when it is static, especially when a person is occluded by more than 50%. However, when the robot is moving along a lawnmower trajectory outside the CCTV’s FOV, it detects the social distancing breaches that could be at any of the 20 locations regardless of occlusions. This is because the robot’s RGB-D camera eventually obtains an unoccluded view as it moves.

We also test the robot-only system’s ability to detect breaches and alert pedestrians in non-laboratory indoor scenes such as lobbies, narrow corridors, etc. These different environments vary in terms of lighting conditions (which affects pedestrian and breach detection) and the available free space for the robot to move. We observe that our system is robust under changes in all the aforementioned factors (see S1 Video).

The robot-CCTV hybrid configuration provides the best performance of the three configurations in terms of detecting novel breaches at the most locations when the robot is static. This is because, when the robot is outside the sensing region of the CCTV camera, the hybrid configuration monitors the largest area in the environment. When the robot is mobile, continuously following a lawnmower path, social distancing breaches in all 40 locations can be detected. This configuration also provides better tracking capabilities when a pedestrian is walking (see subsequent sections). We also note that, when people are static, the robot attends to 100% of the detected breaches while avoiding all the obstacles in the environment.

#### Breach detection accuracy with multiple walking pedestrians

We measure the breach detection accuracy (metric 5) of the robot-CCTV hybrid system in scenario 4 with multiple walking pedestrians. The number of walking pedestrians is varied between two to six for different trials of the experiment and in each trial, the number of breaches is kept constant. Each trial is recorded for 60 seconds (i.e., *T* = 60 in Eq 9) and is repeated for five times and the results are averaged. The results are shown in Table 2. The numbers in the parenthesis in the first column indicate how the total number of pedestrians is split into non-compliant groups as they walk.

**Table 2:** The percentage of breaches detected by the robot-CCTV hybrid setup with different numbers of walking pedestrians.

Total number of pedestrians	Number of non-compliant groups at any instant	Breach detection accuracy (for Robot-CCTV Hybrid)
2	1	89%
3 (2 + 1)	1	88%
4 (2 + 2)	2	89.50%
5 (2 + 3)	2	93%
6 (2 + 2 + 2)	3	89.33%
6 (3 + 3)	2	96.50%

The table shows the effectiveness of the robot-CCTV hybrid system in accurately detecting the actual number of social distancing breaches in the environment over a period of 60 seconds. Each trial is repeated five times and the results are averaged. The first column indicates the total number of pedestrians and how they are split while walking (eg. 2 + 1 indicates two people walking together and one person walking alone). The high breach detection accuracy denotes that our method and setup are effective in detecting the correct number of breaches in the environment.

The breach detection accuracy is a measure of the difference between the number of detected breaches versus the actual number of breaches over a period of time. We observe that for our robot-CCTV hybrid, it is always above 88%, indicating that our method accurately detects the actual number of non-compliant groups. We observed that the detection accuracy was typically affected when the pedestrians moved into blind spots between the sensing regions of the CCTV camera and the robot’s RGB-D camera. Another factor which affected the results was the robot’s pose due to the lawnmower trajectory relative to the pedestrians when they were walking in random directions.

The size of each non-compliant group also affected the overall results (as observed from rows four and six in Table 2). Breaches by bigger groups of people are easier to detect. This is because a breach could be recorded even if a subset of all the group members is visible to the cameras. Moreover, such groups also have a lower chance of being completely occluded by objects in the environment. However, the orientations and the walking speeds of the pedestrians did not affect the breach detection accuracy. Overall, we observe that our robot-CCTV hybrid is effective in accurately detecting the actual number of social distancing breaches in the environment.

#### Computation time and complexity for breach detection

**Fig 8:**
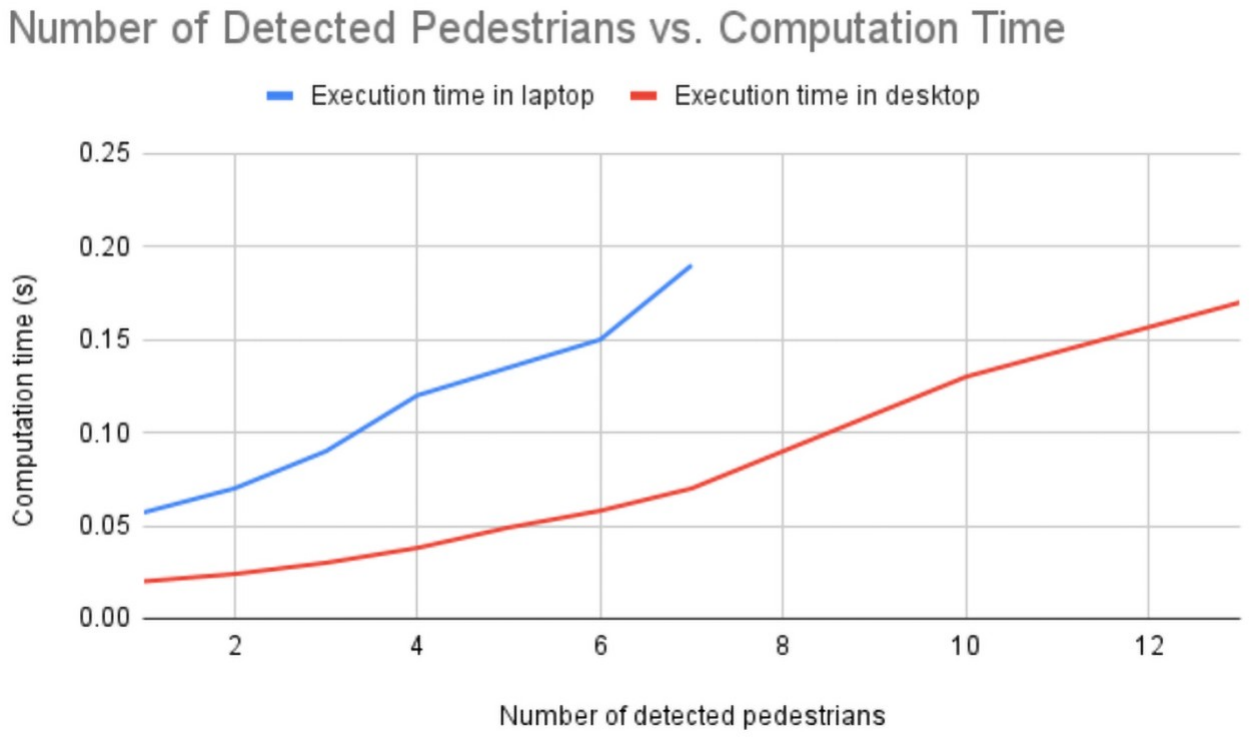
Graphs of our breach detection’s computation time versus the number of detected pedestrians. The graph shows the computation times while running our breach detection implementations for the RGB-D camera on a robot-mounted laptop, and the wall-mounted CCTV camera on a desktop (see Implementation section for specifications). The values were recorded while evaluating in scenario 4 with added human shaped cardboard cutouts to get the total number of detected pedestrians to be 13. We observe that based on the FOV and sensing regions of the two cameras, the corresponding computation times in the laptop and desktop are satisfactory.

We recorded the computation times for detecting breaches with the increase in the number of pedestrians in the environment (scenario 4). To evaluate the computation times when more people than six people are present, we use human shaped cardboard cutouts (to have up to 13 pedestrians). We compare the computation times to detect breaches on the laptop mounted on the robot and the desktop which processes the CCTV images (see Implementation section for their specifications). The breach detection implementations for the two cameras are different as explained in the Methods section and the Implementation section. The results are shown in Fig 8.

We observe that the computation time increases non-linearly with the increase in the number of detected pedestrians. This follows from observing algorithm 1, which has a worst case time complexity of $O(n^{2})$, where n is the number of detected pedestrians. On average, due to the RGB-D camera’s limited FOV and sensing range, the robot-mounted laptop does not detect more than seven pedestrians simultaneously. The CCTV’s larger sensing region allows tens of pedestrians to be detected. For both these cases, from the graph in Fig 8, we observe that the breach detection time is around 0.16 seconds. This is well within the acceptable rate for the robot to enforce social distancing norms even with dynamic pedestrians.

We also observed that since pedestrian and breach detection were not affected by the orientations and velocities of the pedestrians, the time complexity also does not depend on those factors.

#### RGB-D pedestrian tracking duration

In this experiment, we observe the effects of our robot’s angular motion on the duration for which it can track walking pedestrians using only its onboard RGB-D sensor with low FOV. We evaluate the pedestrian tracking time metric in scenario 3. We vary the maximum angular velocity of the robot to measure the differences in tracking performance (Table 3).

**Table 3:** Tracking duration with varying pedestrian velocities.

Case 1: Maximum Robot Angular Velocity = 0.5 rad/sec	
**Pedestrian Velocity (m/sec)**	**Tracking time (sec)**
0.25	20 (20)
0.5	6.59 (10)
0.75	3.15 (6.67)
1	2.95 (5)
Case 2: Maximum Robot Angular Velocity = 0.75 rad/sec	
0.25	20 (20)
0.5	10 (10)
0.75	3.91 (6.67)
1	2.93 (5)
Case 3: Maximum Robot Angular Velocity = 1.0 rad/sec	
0.25	20 (20)
0.5	10 (10)
0.75	6.58 (6.67)
1	2.77 (5)

The duration for which the robot tracks a walking pedestrian for different pedestrian walking speeds and maximum angular velocities of the robot. The pedestrian walks 5 meters in a direction perpendicular to the robot’s orientation and it has to rotate and track the walking pedestrian. The ideal time for which a pedestrian should be tracked is given in the bracket beside the actual time. The robot can effectively track a pedestrian walking at up to 0.75 m/sec when its angular velocity is 1 rad/sec.

As expected, we observe that the greater the maximum angular velocity of the robot, the better it can track a fast-moving pedestrian. However, for high walking speeds (≥ 0.75*m*/*s*) even a very high maximum angular speed (1 rad/s) is insufficient to track a person for the ideal time that the person needs to be tracked (shown in gray in Table 3). This justifies the need for integrating with existing CCTV camera setup for better tracking capabilities.

#### CCTV-guided walking locked pedestrian pursuit

**Fig 9:**
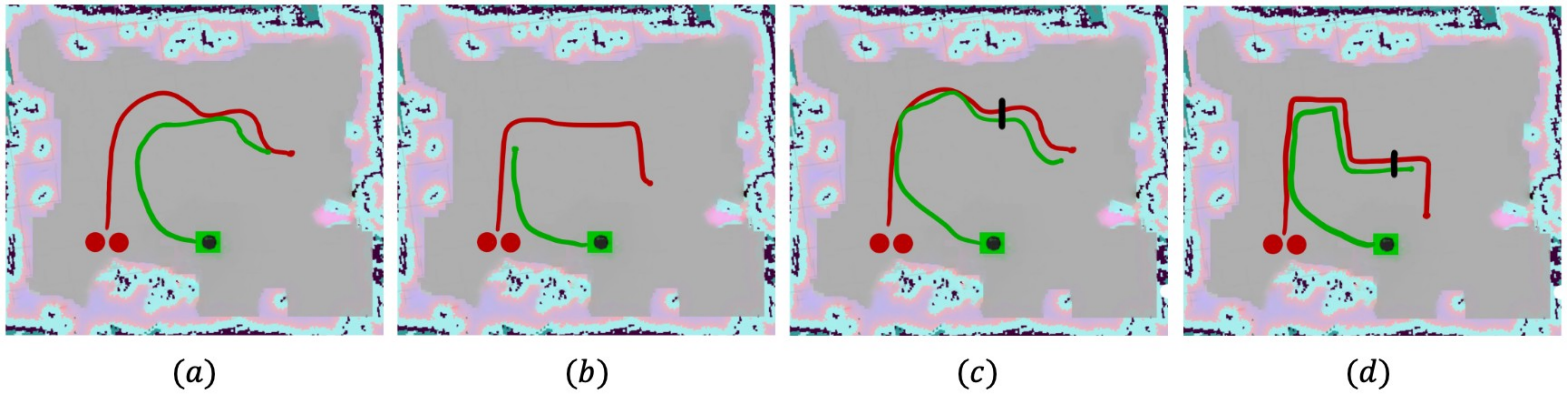
Improved pedestrian tracking using CCTV camera. Trajectories of two non-compliant pedestrians (in red) and the robot pursuing them (in green) in the mapped environment shown in Fig 5c. The pink and blue colors denote the static obstacles in the environment. **a**. The robot only uses its RGB-D camera to track the pedestrian and pursues the pedestrians successfully when they move in a smooth trajectory. **b**. The robot’s RGB-D camera is unable to track the pedestrians when they make a sudden sharp turn. **c**. When the CCTV camera is used to track the pedestrians, the robot follows their trajectories more closely. **d**. Pedestrians making sharp and sudden turns can also be tracked. The black line denotes the point at which the pedestrians leave the CCTV camera’s FOV, and the RGB-D camera tracks the pedestrians from this point. Sharp turns in **d** again become a challenge.

We qualitatively demonstrate how our robot pursues two walking non-compliant pedestrians (scenario 5) by plotting their trajectories in the cases where the RGB-D (see Fig 9a and 9b) or the CCTV camera (see Fig 9c and 9d) detects them. Fig 9a shows that when the pedestrians walk in a smooth trajectory without sharp turns, the robot is able to successfully track them throughout their walk.

In Fig 9b, we observe that when the pedestrians make a sharp turn and manage to go outside the limited FOV of the RGB-D camera, the robot is unable to pursue him/her. The pedestrians were walking at speeds ∼0.75 m/sec. This issue is alleviated when the CCTV camera tracks both the pedestrians instead of the RGB-D camera. Fig 9c and 9d show that the robot is able to track the pedestrians more closely and accurately with the goal data computed using the CCTV’s localization. In addition, sudden and sharp turns by the pedestrians are handled with ease, and pedestrians moving at speeds ∼ 0.75 m/sec can be tracked and pursued, which was not possible with the robot- only configuration. When the pedestrians move out of the CCTV camera’s FOV (black line in Fig 9c and 9d), the data from the robot’s RGB-D camera helps pursue the two pedestrians immediately. However, the pedestrians’ sharp turns again becomes a challenge to track.

## Conclusion

We present a novel method to detect social distancing breaches in indoor scenes using simple sensors such as RGB-D and CCTV cameras. We use a mobile robot to attend to the individuals who are non-compliant with the social distancing norm and to encourage them to move apart by displaying a message on a screen mounted on the robot. We demonstrate our method’s effectiveness in localizing pedestrians, detecting breaches, and pursuing walking pedestrians. We demonstrate that the CCTV+robot hybrid configuration outperforms configurations in which only one of the two components is used for tracking and alerting non-compliant pedestrians.

Our method has a few limitations. For instance, it does not distinguish between strangers and people from the same household. Therefore, all individuals in an indoor environment are encouraged to maintain a 6-foot distance from each other. Our current approach for issuing a warning to violating pedestrians using a monitor has limitations, and we need to develop better human-robot interaction approaches. As more such monitoring robots are used to check for social distances or collect related data, this could also affect the behavior of pedestrians in different settings. We need to perform more studies on the social impact of such robots. Due to COVID restrictions, we have only been able to evaluate the performance of the CS-robot in our low to medium density indoor settings. Eventually, we want to evaluate the robot’s performance in crowded public settings and outdoor scenarios.

We also need to design better techniques to improve the enforcement of social distancing by using better human-robot interaction methods. Improved exploration techniques to minimize the loss of detecting breaches could also be addressed in our future work. We would also like to develop methods for detecting if the people in the robot’s surroundings are wearing masks. Specifically, we would like to formulate methods that are robust to changes in the pose of people’s bodies and heads.

## Supporting information

S1 Video Video with real-world demonstrations. The attached supporting video shows how our robot detects and enforces social distancing breaches using its on-board RGB-D camera, and using an assistive CCTV camera in a laboratory setting. It also demonstrates our robot executing lawnmower trajectories to explore its environment and detect new non-compliant groups (both static and dynamic) and pursue them until they observe the social distancing norms.
(MP4)

## References

[1] Mao L. Agent-based simulation for weekend-extension strategies to mitigate influenza outbreaks. BMC Public Health. 2011;11(1):522. doi: 10.1186/1471-2458-11-522 21718518 PMC3146865

[2] Kumar S, Grefenstette JJ, Galloway D, Albert SM, Burke DS. Policies to reduce influenza in the workplace: impact assessments using an agent-based model. Am J Public Health. 2013;103(8):1406–1411. doi: 10.2105/AJPH.2013.301269 23763426 PMC3893051

[3] Timpka T, Eriksson H, Holm E, Stromgren M, Ekberg J, Spreco A, et al. Relevance of workplace social mixing during influenza pandemics: an experimental modelling study of workplace cultures. Epidemiol Infect. 2016;144(10):2031–2042. doi: 10.1017/S0950268816000169 26847017 PMC9150599

[4] Milne GJ, Kelso JK, Kelly HA, Huband ST, McVernon J. A small community model for the transmission of infectious diseases: comparison of school closure as an intervention in individual-based models of an influenza pandemic. PLoS ONE. 2008;3(12):1–7. doi: 10.1371/journal.pone.0004005 19104659 PMC2602849

[5] Cauchemez S, Valleron AJ, Boëlle PY, Flahault A, Ferguson NM. Estimating the impact of school closure on influenza transmission from Sentinel data. Nature. 2008;452(7188):750–754. doi: 10.1038/nature06732 18401408

[6] Nguyen CT, Mulya Saputra Y, Van Huynh N, Nguyen NT, Viet Khoa T, Tuan BM, et al. Enabling and Emerging Technologies for Social Distancing: A Comprehensive Survey. arXiv e-prints. 2020; p. arXiv:2005.02816.

[7] Yang D, Yurtsever E, Renganathan V, Redmill K, Ozguner U. A Vision-based Social Distancing and Critical Density Detection System for COVID-19; 2020. 10.3390/s21134608 PMC8271503 34283141

[8] Singh Punn N, Sonbhadra SK, Agarwal S. Monitoring COVID-19 social distancing with person detection and tracking via fine-tuned YOLO v3 and Deepsort techniques. arXiv e-prints. 2020; p. arXiv:2005.01385.

[9] Ghodgaonkar I, Chakraborty S, Banna V, Allcroft S, Metwaly M, Bordwell F, et al. Analyzing Worldwide Social Distancing through Large-Scale Computer Vision. arXiv e-prints. 2020; p. arXiv:2008.12363.

[10] Murphy RR, Babu Manjunath Gandudi V, Adams J. Applications of Robots for COVID-19 Response. arXiv e-prints. 2020; p. arXiv:2008.06976.

[11] Fan T, Chen Z, Zhao X, Liang J, Shen C, Manocha D, et al. Autonomous Social Distancing in Urban Environments using a Quadruped Robot. arXiv e-prints. 2020; p. arXiv:2008.08889.

[12] Chen YF, Liu M, Everett M, How JP. Decentralized non-communicating multiagent collision avoidance with deep reinforcement learning. In: ICRA. IEEE; 2017. p. 285–292.

[13] Everett M, Chen YF, How JP. Motion planning among dynamic, decision-making agents with deep reinforcement learning. In: IROS. IEEE; 2018. p. 3052–3059.

[14] Fox D, Burgard W, Thrun S. The dynamic window approach to collision avoidance. IEEE Robotics Automation Magazine. 1997;4(1):23–33. doi: 10.1109/100.580977

[15] Van Den Berg J, Ming Lin, Manocha D. Reciprocal Velocity Obstacles for real-time multi-agent navigation. In: 2008 IEEE International Conference on Robotics and Automation; 2008. p. 1928–1935.

[16] Van Den Berg J, Guy S, Lin M, Manocha D. Reciprocal n-body collision avoidance. In: Robotics Research—The 14th International Symposium ISRR. No. STAR in Springer Tracts in Advanced Robotics; 2011. p. 3–19.

[17] Long P, Fan T, Liao X, Liu W, Zhang H, Pan J. Towards Optimally Decentralized Multi-Robot Collision Avoidance via Deep Reinforcement Learning. arXiv e-prints. 2017; p. arXiv:1709.10082.

[18] Liang J, Patel U, Jagan Sathyamoorthy A, Manocha D. Realtime Collision Avoidance for Mobile Robots in Dense Crowds using Implicit Multi-sensor Fusion and Deep Reinforcement Learning. arXiv e-prints. 2020; p. arXiv:2004.03089.

[19] Sathyamoorthy AJ, Liang J, Patel U, Guan T, Chandra R, Manocha D. DenseCAvoid: Real-time Navigation in Dense Crowds using Anticipatory Behaviors. arXiv e-prints. 2020; p. arXiv:2002.03038.

[20] Liang J, Qiao YL, Manocha D. OF-VO: Reliable Navigation among Pedestrians Using Commodity Sensors. arXiv e-prints. 2020; p. arXiv:2004.10976.

[21] Sathyamoorthy AJ, Patel U, Guan T, Manocha D. Frozone: Freezing-Free, Pedestrian-Friendly Navigation in Human Crowds. IEEE Robotics and Automation Letters. 2020;5(3):4352–4359. doi: 10.1109/LRA.2020.2996593

[22] Wojke N, Bewley A, Paulus D. Simple online and realtime tracking with a deep association metric. In: 2017 IEEE International Conference on Image Processing (ICIP); 2017. p. 3645–3649.

[23] Redmon J, Farhadi A. YOLOv3: An Incremental Improvement. CoRR. 2018;abs/1804.02767.

[24] Sathyamoorthy AJ, Patel U, Ajay Savle Y, Paul M, Manocha D. COVID-Robot: Monitoring Social Distancing Constraints in Crowded Scenarios. arXiv e-prints. 2020; p. arXiv:2008.06585. 10.1371/journal.pone.0259713 PMC8635356 34851982

